# Influence mechanism of the temporal duration of laser irradiation on photoacoustic technique: a review

**DOI:** 10.1117/1.JBO.29.S1.S11530

**Published:** 2024-04-17

**Authors:** Rongkang Gao, Yan Liu, Sumin Qi, Liang Song, Jing Meng, Chengbo Liu

**Affiliations:** a Chinese Academy of Sciences, Shenzhen Institute of Advanced Technology, Research Center for Biomedical Optics and Molecular Imaging, Key Laboratory of Biomedical Imaging Science and System, Shenzhen, China; b Qufu Normal University, School of Cyberspace Security, Qufu, China

**Keywords:** pulse duration, stress relaxation, thermal relaxation, thermal diffusion, center frequency, nonlinear effect

## Abstract

**Significance:**

In the photoacoustic (PA) technique, the laser irradiation in the time domain (i.e., laser pulse duration) governs the characteristics of PA imaging—it plays a crucial role in the optical-acoustic interaction, the generation of PA signals, and the PA imaging performance.

**Aim:**

We aim to provide a comprehensive analysis of the impact of laser pulse duration on various aspects of PA imaging, encompassing the signal-to-noise ratio, the spatial resolution of PA imaging, the acoustic frequency spectrum of the acoustic wave, the initiation of specific physical phenomena, and the photothermal–PA (PT-PA) interaction/conversion.

**Approach:**

By surveying and reviewing the state-of-the-art investigations, we discuss the effects of laser pulse duration on the generation of PA signals in the context of biomedical PA imaging with respect to the aforementioned aspects.

**Results:**

First, we discuss the impact of laser pulse duration on the PA signal amplitude and its correlation with the lateral resolution of PA imaging. Subsequently, the relationship between the axial resolution of PA imaging and the laser pulse duration is analyzed with consideration of the acoustic frequency spectrum. Furthermore, we examine the manipulation of the pulse duration to trigger physical phenomena and its relevant applications. In addition, we elaborate on the tuning of the pulse duration to manipulate the conversion process and ratio from the PT to PA effect.

**Conclusions:**

We contribute to the understanding of the physical mechanisms governing pulse-width-dependent PA techniques. By gaining insight into the mechanism behind the influence of the laser pulse, we can trigger the pulse-with-dependent physical phenomena for specific PA applications, enhance PA imaging performance in biomedical imaging scenarios, and modulate PT-PA conversion by tuning the pulse duration precisely.

## 1 Introduction

Photoacoustic (PA) imaging has emerged as a promising medical imaging technology.^1^^–^^5^ By converting optical energy into acoustic energy, which is two to three orders of magnitude less scattered than photons, PA imaging surpasses the optical diffusion limit of 1 mm in biological tissue and significantly extends the imaging depth to ranges that are inaccessible by conventional optical imaging techniques.^1^^,^^6^^–^^12^ Unlike conventional ultrasound imaging methods that rely on mechanical properties for contrast generation, PA imaging retains the rich optical absorption contrast derived from photon absorption.^5^^,^^13^^,^^14^ These distinctive features make PA imaging a revolutionary modality with broad applications in biomedical research.^7^^,^^15^^–^^26^ PA imaging is based on the PA effect, wherein biological tissues are illuminated by a nonionizing short-pulsed laser beam or rapidly modulated radiation as probing energy.^1^^,^^3^^,^^4^^,^^27^^–^^32^ The illumination induces temporally confined optical absorption, which converts into heat, causing a transient local temperature increase. This increase in temperature leads to thermal-elastic expansion, resulting in an initial pressure rise that propagates as wideband frequency ultrasonic waves. These waves are detectable by an ultrasound transducer (UST).^33^^–^^35^

**Fig. 1:**
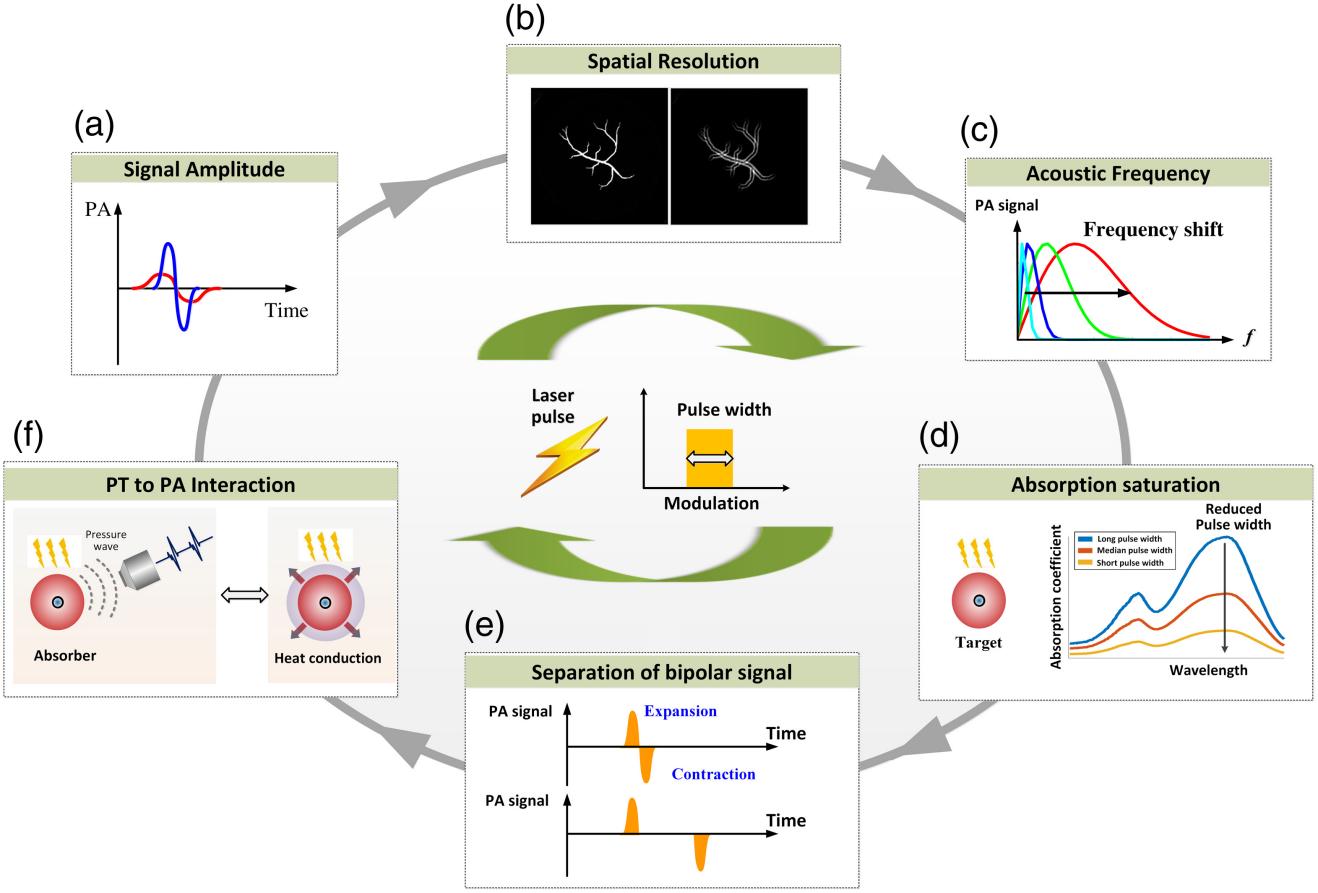
Principle illustration of the influence of the laser pulse duration on various aspects, including (a) the SNR of PA signals, (b) the spatial resolution of PA images, (c) the acoustic frequency of PA signals, (d) the saturation effect of absorption, (e) the decoupling of bipolar PA signals, and (f) the PT-PA conversion.

The interaction of laser radiation in the time domain with the thermoelastic vibration of tissue can be quite complex. The laser pulse duration governs the type of laser–tissue interaction that may occur and dominates the transfer of heat energy to the mechanical vibration mode.^35^ This affects the signal generation in the optical zone of the tissue as well as the imaging performance of the PA technique. The pulse duration also influences physical phenomena occurrence such as the absorption saturation and the decoupling of the bipolar PA signals. In this paper, we aim to elucidate the underlying mechanisms through which the laser pulse width affects the characteristics of the PA signals in the aforementioned aspects, as depicted in Fig. 1. Distinguished from existing PA reviews, this paper represents the first comprehensive analysis focusing on the physical mechanisms governing pulse-width-dependent PA techniques and offers insights into the behavior of the PA signal and imaging in relation to the laser pulse width.

This paper is structured as follows. Section 2 introduces the theoretical background of PA signal generation, emphasizing the critical time scales associated with radiation exposure. In Sec. 3, we delve into the impact of laser pulse duration on the PA signal amplitude (Sec. 3.1) and its correlation with the lateral resolution of PA imaging (Sec. 3.2). Furthermore, we elaborate on the dependence of the axial resolution of PA imaging on the pulse duration with consideration of the acoustic frequency spectrum (Sec. 3.3). We also analyze the manipulation of the pulse duration to induce absorption-based nonlinearity (Sec. 3.4) and examine the modulation of the pulse duration to separate correlated bipolar PA signals as well as its relevant applications (Sec. 3.5). In addition, we investigate how the duration of radiation exposure can be tuned to manipulate the conversion process and ratio from the photothermal (PT) to PA effect (Sec. 3.6). Finally, Sec. 4 draws concluding remarks.

## 2 Principles

The generation and propagation of the induced acoustic signals are characterized by the following relation:

$$(\nabla^{2}-\frac{1}{v_{s}^{2}}\frac{\partial }{\partial t^{2}})p(r,t)=-\frac{\beta }{C_{P}}\frac{\partial H}{\partial t}.$$ (1)

The relationship between wave propagation (left term) and the source term (right term) is illustrated in Eq. (1), where the source term corresponds to the derivative of the heating function (∂H/∂t). This equation demonstrates that static heating resulting from light exposure does not produce a PA pressure wave. PA signals are exclusively generated by time-varying light exposure. Consequently, incandescent light sources or continuous wave (CW) laser sources do not elicit PA waves, whereas time-modulated and pulsed laser sources do. This observation also explains why the PA effect was first observed using a rapidly interrupted beam of light radiation by Alexander Graham Bell in 1880,^36^ rather than with continuous, steady illumination such as that from an incandescent light bulb. The temporally confined optical absorption leads to thermal-elastic expansion, thereby resulting in PA pressure rises. The PA signal detected by a UST is given as

$$\mathrm{PA}=k\Gamma \eta_{\mathrm{th}}\mu_{a}F,$$ (2)

where $\mu_{a}$ denotes the absorption coefficient of the imaging target, F represents the local optical fluence, $\eta_{\mathrm{th}}$ signifies the coefficient for light-to-heat conversion, Γ is the Grüeneisen parameter, and k denotes the detection sensitivity. Equation (2) provides a quantitative expression for the PA signals under thermal and stress confinements.^4^^,^^37^ In other words, the duration of radiation exposure must be significantly smaller than the thermal and stress relaxation time^4^^,^^37^ for Eq. (2) to be applicable. The thermal relaxation time ($\tau_{\mathrm{th}}$) describes the thermal diffusion of the voxel of interest and is expressed as^37^

$$\tau_{\mathrm{th}}=d_{c}^{2}/\alpha_{\mathrm{th}},$$ (3)

where $d_{c}$ and $\alpha_{\mathrm{th}}$ represent the characteristic dimension of the heated region and the thermal diffusivity, respectively. The stress relaxation time ($\tau_{s}$) characterizes the propagation of pressure within the voxel of interest and is given as^37^

$$\tau_{s}=d_{c}/c,$$ (4)

where c denotes the speed of sound. Table 1 presents the typical thermal and stress relaxation time scales of soft tissue with different characteristic dimensions.

**Table 1:** Typical thermal and stress relaxation time of soft tissue with different characteristic dimensions.

Type of time scale	Different characteristic dimensions		
	$d_{c}=15\;\mu m$	$d_{c}=5\;\mu m$	$d_{c}=1\;\mu m$
$\tau_{\mathrm{th}}$ ^a^	1.7 ms	192 μs	7.6 μs
$\tau_{s}$	10 ns	3.3 ns	0.6 ns

a The thermal diffusivity $\alpha_{\mathrm{th}}$ in the expression of $\tau_{\mathrm{th}}$ [Eq. (3)] is estimated as $1.3\times 10^{-3}\;{\mathrm{cm}}^{2}/s$ for soft tissue according to Ref. 37.

Notably, the stress relaxation time is typically more restrictive than the thermal relaxation time.^37^ This ensures that the built-up heating energy is converted into vibration emission (PA wave) before the heat is transmitted to the neighboring molecules. This is a fortunate prerequisite and can even be considered a condition carefully choreographed by nature because it forms the fundamental mechanism of PA signal generation. If the two time scales occur in a reversed sequence, the conversion from photons to acoustic energy would be hard to accomplish because the optical-absorption-induced heat would be first transferred to adjacent structures/molecules via heat conduction. The energy is considerably dissipated away and released when it reaches the stress relaxation time and would be hard to be converted into acoustic energy via stress expansion due to the lack of sufficient energy density for PA generation.

## 3 Influencing Aspects

### 3.1 Signal Amplitude (Signal-to-Noise Ratio)

**Fig. 2:**
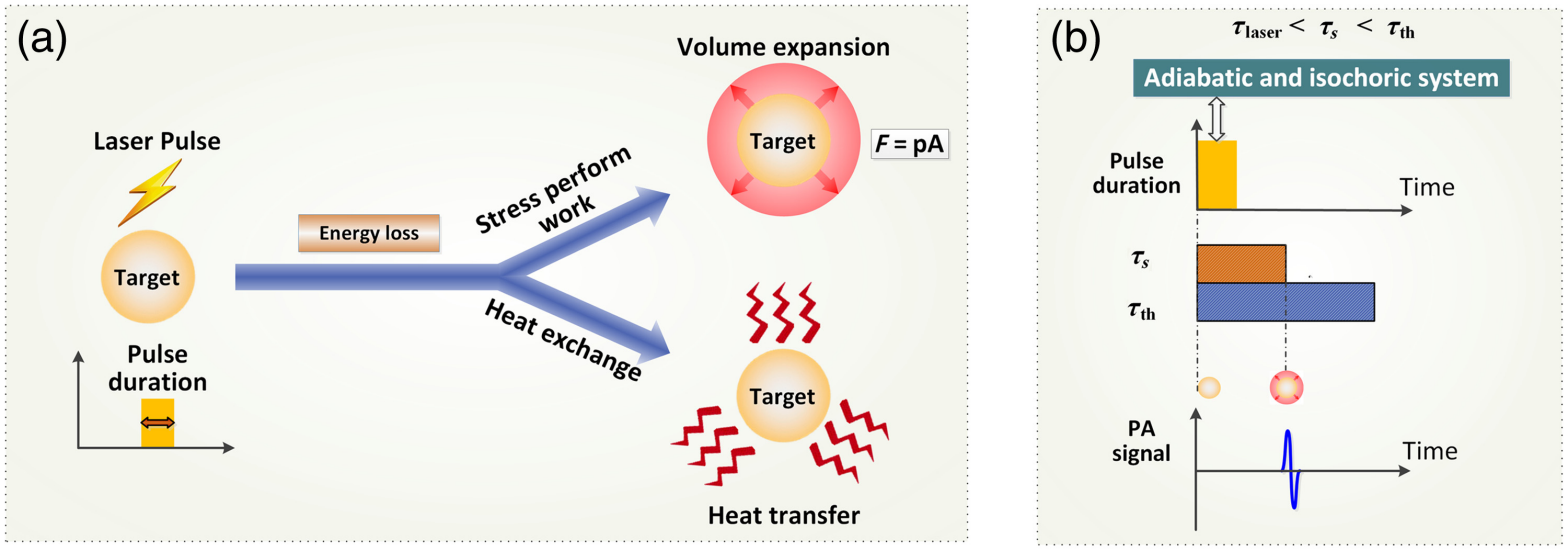
Principle illustration of the influence of the laser pulse duration on the signal-to-noise ratio. (a) Dual mechanisms of energy dissipation of optical absorption. (b) Adiabatic and isochoric processes occurring within the pulse duration interval that fulfills both stress and thermal confinement criteria.

The reduction in the optical excitation duration helps guarantee that the optical absorption zone behaves as an adiabatic and isochoric system during the pulse duration (Fig. 2). Separately speaking, when the stress confinement is met, no stress propagation and no deformation of the specific optical absorption zone take place during the pulse duration. Otherwise (the stress confinement is not met), volume expansion occurs and stress begins to perform work, leading to the dissipation of the accumulated energy [Fig. 2(a), top] during the pulse duration. When the thermal confinement is met, the generated heat does not transfer from the optical absorption zone to its neighboring molecules during the pulse width [Fig. 2(a), bottom]. In other words, no heat exchange occurs between the optical absorption zone and its neighboring molecules. These conditions indicate that all of the heat converted by optical absorption is used solely to raise the temperature of the region of photon deposition (i.e., the thermal diffusion and pressure relaxation are negligible during laser illumination). Consequently, the conversion of optical energy deposition to the subsequent instantaneous pressure rise is maximized when it reaches the stress relaxation time [Fig. 2(b)], thereby enhancing the signal-to-noise ratio (SNR) of PA imaging.

**Fig. 3:**
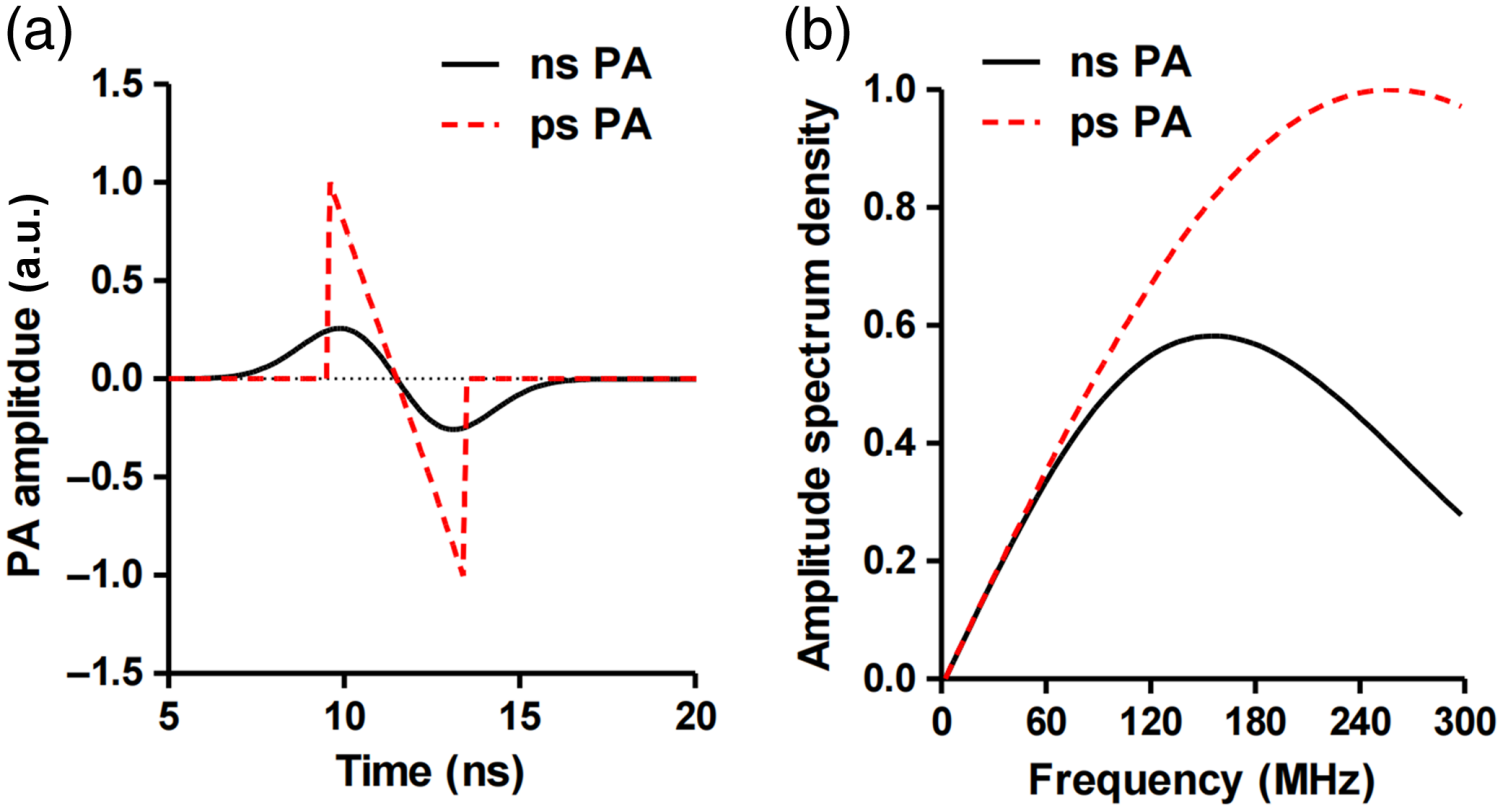
Influence of laser pulse duration on the PA signal amplitude. The simulated PA signals from a 3-μm-diameter sphere excited using laser excitation with a duration of 3 ns and 3 ps in the (a) time and (b) space domains. The PA signals are generated with the same pulse energy. The results show that the short laser pulse yields a higher signal amplitude in both the time and frequency domains. Figures reproduced with permission from Ref. 38.

Figure 3 shows the simulated PA signals excited by a laser pulse with a duration of 3 ns and 3 ps. The PA signals are generated with the same pulse energy. As can be observed in Fig. 3, lasers with a shorter duration (ps) yield a higher signal amplitude in both the time [Fig. 3(a)] and frequency [Fig. 3(b)] domains. The picosecond excitation is more efficient in generating PA signals. For a long duration of laser pulse that violates the thermal or stress confinement, there would still be PA signals but with a low SNR due to the loss of energy. In such cases, the PA signal does not conform to the quantitative relationship characterized by Eq. (2). To address this issue, Newton’s law of cooling has been employed in previous Refs. 39 and 40 to account for the concurrent heat accumulation and diffusion during the laser pulse duration.

### 3.2 Lateral Resolution

**Fig. 4:**
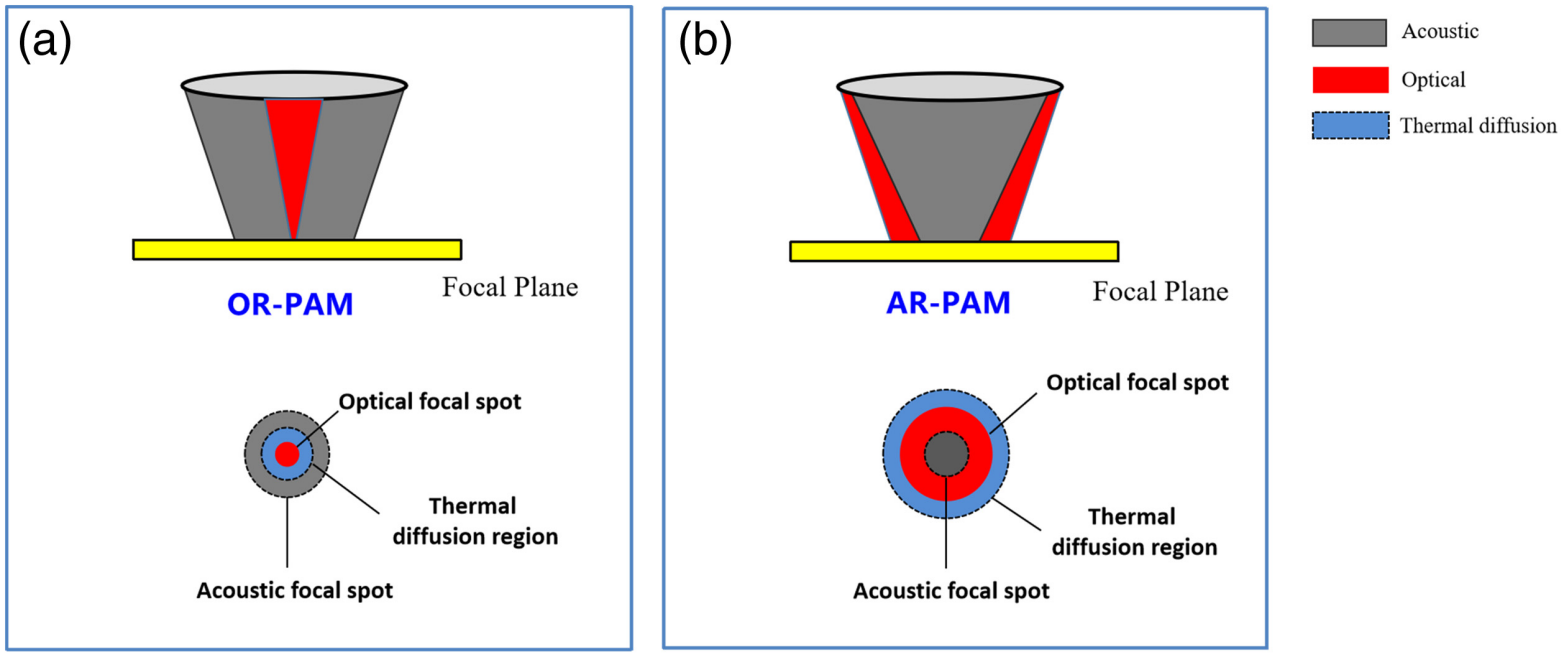
Impact of laser pulse duration on the lateral resolution of both (a) OR-PAM and (b) AR-PAM. The figure illustrates the relative size of the optical focus (red spot), the thermal diffusion region (blue spot), and the acoustic focus (gray spot).

Apart from the SNR, the pulse width also influences the lateral resolution of PA imaging. This section provides a correlation between the laser pulse width in the time domain and the lateral resolution in the spatial domain. Taking the optical-resolution PA microscopy (OR-PAM)^38^^,^^41^^–^^44^ as an example, when the thermal confinement is violated, the optical absorption zone starts transferring the accumulated heat to its neighboring molecules within the pulse duration.^39^^,^^40^ As a result, apart from the optical absorption zone [Fig. 4(a), red region], the neighboring structures outside the illuminated region also bring up the thermoelastic expansion vibration and thus generate the PA signals [Fig. 4(a), blue region]. This phenomenon is further elaborated subsequently in this section. As a result, this effect decreases the lateral resolution of OR-PAM—the lateral resolution ($R_{\text{lateral}}$) is no longer defined by the optical diffraction limit (0.5 λ/NA, where λ and NA denote the optical wavelength and numerical aperture of the objective, respectively) but is influenced by the broadened region of thermal diffusion caused by heat conduction [Fig. 4(a), blue region]. This indicates that $R_{\text{lateral}}$ of OR-PAM is dependent on both the laser pulse duration (time domain) and the laser spot size (spatial domain). As stated in Ref. 45, the long duration of the laser pulse limits the $R_{\text{lateral}}$ to a few hundreds of micrometers, which is far from the micron-scale level of $R_{\text{lateral}}$ in typical OR-PAM that use nanosecond laser pulses.^46^^–^^50^ Consequently, the relationship between the pulse duration and the relaxation time scales governs the mechanism of optical-acoustic interaction and the PA imaging performance.

**Fig. 5:**
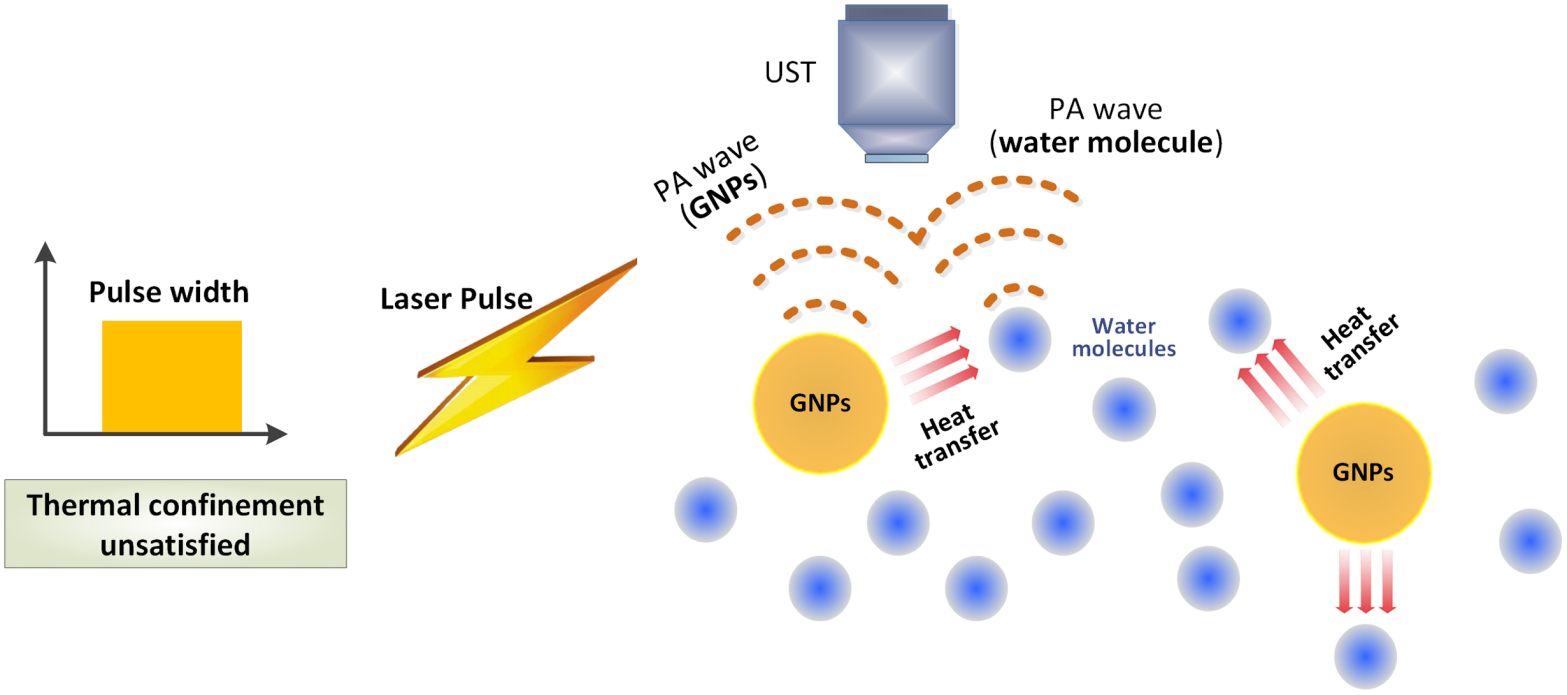
“Borrowed heat energy” of neighboring molecules next to the absorbing molecules also brings up PA signals.

In general, the acoustic wave in PA imaging is produced by the heat induced by light absorption. However, in practice, acoustic waves can be generated by transient heat induced via various means, such as optical absorption or heat conduction. For example, when the thermal confinement is not met, the thermal diffusion region extends beyond the optical absorption zone because of heat conduction. The “borrowed heat energy” of the neighboring structure next to the laser exposure region (optical zone) also brings up the thermoelastic expansion vibration and thus the PA signals. In other words, the molecules that do not absorb photons also generate PA signals. This justifies the fact that the PA signal also comes from the water molecules when imaging nanoparticles solution, such as gold nanoparticles (GNPs),^51^^–^^56^ as depicted in Fig. 5. Due to the small diameter and high thermal conductivity of GNPs, the thermal confinements of GNPs are usually not met because $\tau_{\mathrm{th}}<\tau_{\text{laser}}$. The thermal relaxation time ($\tau_{\mathrm{th}}$) for GNPs with a 100 and a 50 nm diameter is estimated to be $7.9\times 10^{-11}$ and $1.9\times 10^{-11}\;s$, respectively, which are far less than the pulse width ($\tau_{\text{laser}}$) of nanosecond lasers. The thermal relaxation time $\tau_{\mathrm{th}}$ of GNPs was estimated according to Eq. (3) ($\alpha_{\mathrm{th}}=127\;{\mathrm{mm}}^{2}/s$^57^). The generation of PA signal from the surrounding media^58^^,^^59^ (i.e., water, if a GNP solution is used) is due to the occurrence of the thermal exchange between GNPs and water (Fig. 5) within the time scale of $\tau_{\text{laser}}$. The ratio of ${\mathrm{PA}}_{\text{surrounding}}$ to ${\mathrm{PA}}_{\mathrm{GNPs}}$ is dependent on the relative magnitude between $\tau_{\mathrm{th}}$ and $\tau_{\text{laser}}$. This scenario is applicable to different imaging objectives when the thermal confinement is unsatisfied.

The laser pulse width could also influence the spatial resolution of other PA imaging modalities, such as the lateral resolution in acoustic-resolution PA microscopy (AR-PAM)^60^^–^^73^ and PA computed tomography (PACT),^74^^–^^86^ but the influencing mechanism is different compared with OR-PAM. Due to the employment of diffuse optical illumination, the region of the optical excitation is larger than that of the acoustic detection. In such cases, the broadened thermal diffusion region relative to the optical illumination induced by heat conduction [Fig. 4(b), blue region] does not influence the acoustic detection region [Fig. 4(b), gray region] or the acoustic resolution. However, the pulse duration influences the lateral resolution of AR-PAM and PACT via the modulation of the acoustic center frequency ($f_{c}$)—the increase of the laser pulse duration downshifts the acoustic frequency spectrum of the pressure wave, as is elaborated in Sec. 3.3. In AR-PAM, the lateral resolution is given as $R_{\text{lateral}}=0.71c/f_{c}NA$, where c is the speed of sound and NA is the numerical aperture of the UST. Thus, a higher center frequency leads to a finer resolution. In such cases, a long pulse duration causes a decreased resolution of the PA images because it triggers the low-frequency content of the acoustic wave.^87^ In PACT, the lateral resolution is determined by both the bandwidth of the detected signal and the detector aperture.^88^ Note that a short laser pulse (e.g., several ns or smaller) can trigger high-frequency acoustic waves to hundreds of megahertz or higher [Fig. 3(b)], but the UST generally is only able to detect a portion of the frequency spectrum due to its limited bandwidth and center frequency (generally tens of MHz). Thus, for short laser pulses, the center frequency and bandwidth of the UST are more restrictive, thereby limiting the lateral resolution of AR-PAM and PACT. Conversely, for long laser pulses (e.g., tens of ns or longer), the lateral resolution is influenced by the pulse duration because a long laser pulse may trigger an acoustic wave with a lower center frequency and narrower bandwidth compared with that of the UST.

The effect of pulse duration on the lateral resolution was exemplified in Ref. 68 in which an intensity-modulated CW laser diode was applied to the system of AR-PAM. The corresponding lateral resolution (0.45 mm) is considerably lower than that of the typical AR-PAM system using a nanosecond-pulsed laser, where $R_{\text{lateral}}$ is generally around tens of microns.^1^^,^^73^^,^^89^^–^^91^

### 3.3 Axial Resolution

**Fig. 6:**
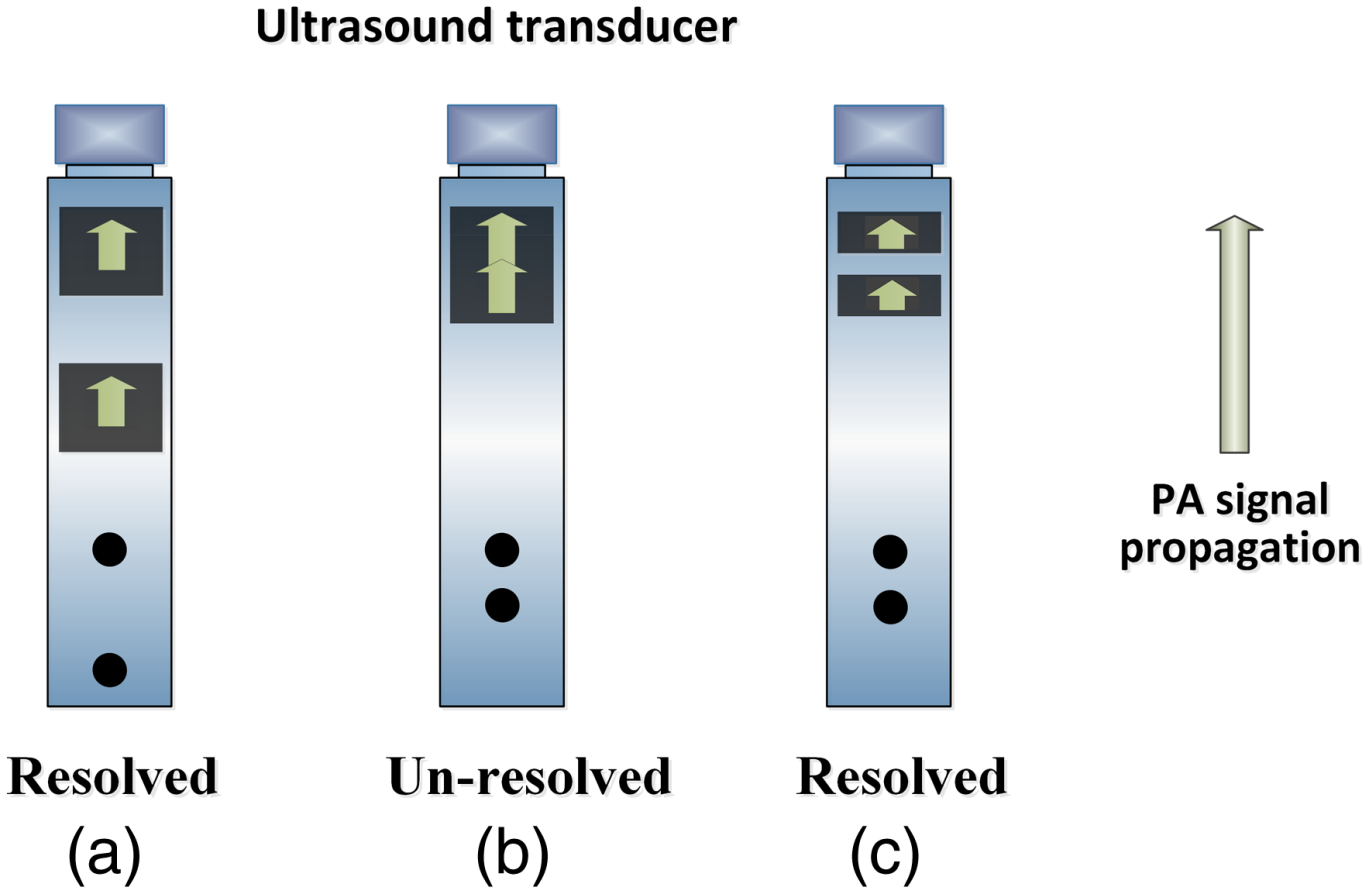
Dependence of the axial resolution of PA imaging on the temporal duration of PA signals. (a) The two absorbers can be resolved when the corresponding PA signals are well separated and arrive at the UST individually. (b) When closely positioned, the two absorbers cannot be resolved because the two PA signals overlap. (c) The two absorbers can still be resolved as the PA signal duration becomes smaller, which corresponds to a finer axial resolution.

This section analyzes the relationship between the pulse width and the axial resolution of PA imaging with consideration of the acoustic frequency spectrum of the pressure wave.^87^^,^^92^ In PA imaging, the acoustic resolution, whether in the context of PAM or PACT, is determined by the duration of the detected acoustic wave. As depicted in Fig. 6, the two absorbers [Fig. 6(a)] can be distinguished by the UST because their corresponding PA signal is sufficiently spaced apart in the time domain and arrives at the UST separately. However, when the two absorbers are closer together [Fig. 6(b)], they cannot be resolved by the UST because their corresponding PA signals overlap in the time domain. By shortening the duration of the acoustic waves in the time domain [Fig. 6(c)], these two absorbers can still be distinguished even if they are closely spaced. According to the time-frequency transform, the duration of the pulse corresponds to the bandwidth of the acoustic wave in the frequency domain. The bandwidth is approximately proportional to the center frequency ($f_{c}$) of the acoustic wave.

The duration of the laser exposure affects the axial resolution of PA imaging by altering the center frequency (and the bandwidth of the frequency spectra) of the optically induced acoustic wave. As the laser pulse duration increases, the center frequency ($f_{c}$) decreases. This mechanism can be comprehended by tracing the historical development of the PA technique. The physical basis of PA imaging, known as the PA effect, was discovered by Alexander Graham Bell in 1880.^36^ In his work, a rapidly interrupted beam of light energy was used to induce acoustic waves. The duration of radiation exposure in such cases was considerably long compared with the advanced pulsed laser sources (e.g., nanosecond and femtosecond lasers) used in modern PA techniques. Consequently, the emitted acoustic waves exhibit a low center frequency (<20  kHz) within the range detectable by the human ear. With the emergence of advanced pulsed laser sources (nanosecond and femtosecond lasers) employed for optical excitation, the center frequency of the generated pressure wave has significantly increased, reaching the magnitude of MHz.^1^^–^^5^

The influence of the pulse duration on the center frequency of the pressure wave can be also explained from the perspective of signal convolution. The detected PA waveform in the time domain can be described as the convolution of the imaging target structure, the laser pulse duration, and the UST’s impulse response,^93^ given as

**Fig. 7:**
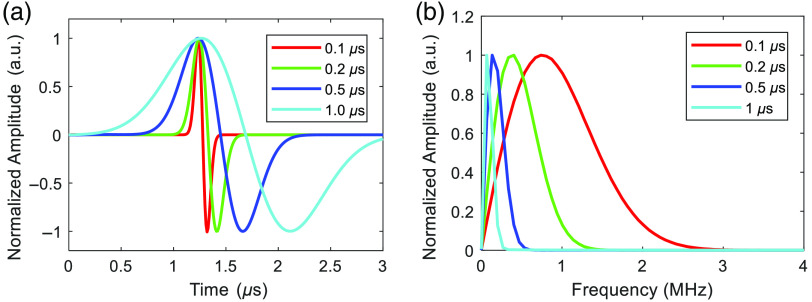
Dependence of PA signals on the laser pulse duration in the (a) time and (b) frequency domains. The simulated PA signals of a-25-μm-diameter sphere were excited by various laser pulse durations ranging from 0.1 to 1  μs, as shown by different colors in the legend. These simulations were performed using the k-wave toolbox.

**Fig. 8:**
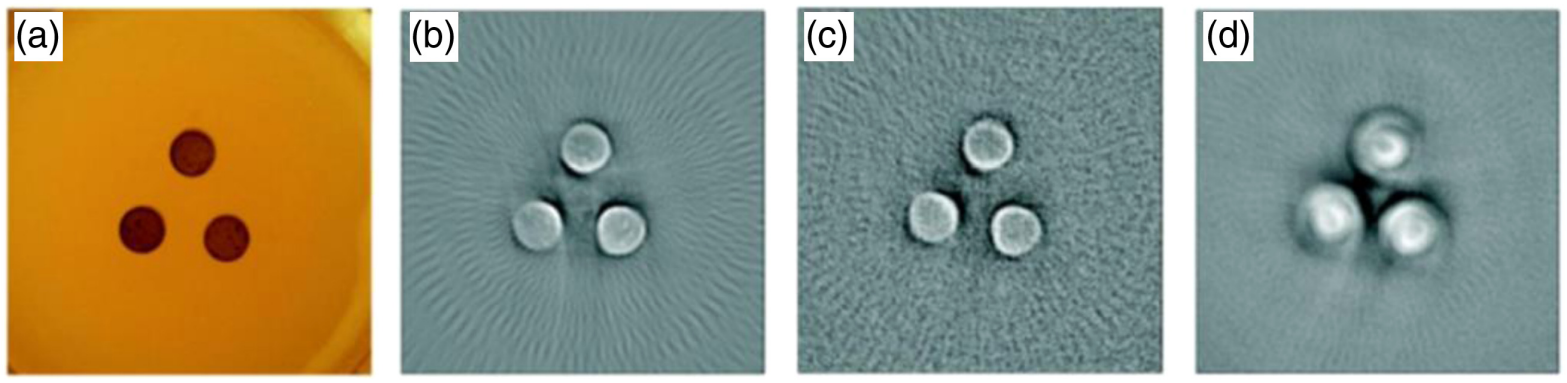
Impacts of laser pulse duration on spatial resolution. (a) The photograph of the imaging objective. (b)–(d) The PA imaging results obtained using laser excitation with a duration of 7, 65, and 500 ns, respectively. Lasers with shorter durations yield sharper images and better resolutions. Figures reproduced with permission from Ref. 94.

P(t)=S*L(t)*UST(t), (5)

where the symbol * denotes the convolution operation, P(t) indicates the PA waveform in the time domain, S denotes the imaging target, and L(t) and UST(t) represent the laser pulse and UST impulse response in the time domain, respectively. Therefore, a long duration of radiation exposure L(t) leads to a broadening of the induced PA signal P(t) in the time domain. According to the time-frequency transformation, this results in a narrower frequency spectrum P(f) and a lower center frequency ($f_{c}$) of the PA signals. Figure 7 illustrates the effect of laser pulse duration on the PA signals in the time and frequency domains, where the simulated PA signals were excited by various laser pulse durations that range from 0.1 to 1  μs. The results are consistent with our analysis—an increased pulse duration broadens the PA profile in the time domain [Fig. 7(a)] and narrows it in the frequency domain [Fig. 7(b)].^87^ The simulation in Fig. 3 shows a similar tendency of the PA signal in the time and frequency domains with respect to the laser pulse duration. Notably, the picosecond excitation is more effective in generating high-frequency signals (>180  MHz), as shown in Fig. 3(b). For experimental validation, Ref. 94 evaluated the spatial resolution of PA images as a function of pulse duration, as illustrated in Fig. 8. This study utilized a UST to perform a circular scan and employed the back-projection algorithm for two-dimensional image reconstruction, which is equivalent to a circular array PACT system. The results demonstrate that prolonged radiation duration yields blurred images with reduced resolution, consistent with our theoretical analysis.

A laser diode source with a shorter pulse duration (e.g., ns and ps scales) would be preferred because it produces a high-frequency content that lies within a broad bandwidth of UST. It is worth mentioning that the center frequency ($f_{c}$) of the detected PA signal is determined by four factors: (1) the width of the laser pulse, (2) the desired tissue depth for imaging, (3) the size of the imaging target, and (4) the frequency response of the UST. Although tuning the laser pulse width can achieve high-frequency acoustic waves, the UST can only receive a portion of the frequency spectrum due to its limited bandwidth. For short laser pulses (e.g., several ns or smaller), as discussed in Sec. 3.2, the bandwidth of the UST is typically narrower than that of the generated acoustic wave. Thus, the UST directly determines the axial resolution. By contrast, for long laser pulses (e.g., tens of ns or longer), the axial resolution is considerably influenced by the pulse duration because a long laser pulse may cause a narrower bandwidth compared with that of the UST. Moreover, biological tissues attenuate ultrasound waves in a frequency-dependent manner, with high-frequency components experiencing greater attenuation compared with low-frequency components. As a result, an improved axial resolution can be made by optimizing the PA wave generation and the frequency response of the UST: the former by selecting the laser pulse duration in relation to the depth of the target in biological tissues and the latter by matching the bandwidth of the UST to that of the generated acoustic wave.

### 3.4 Absorption Saturation

**Fig. 9:**
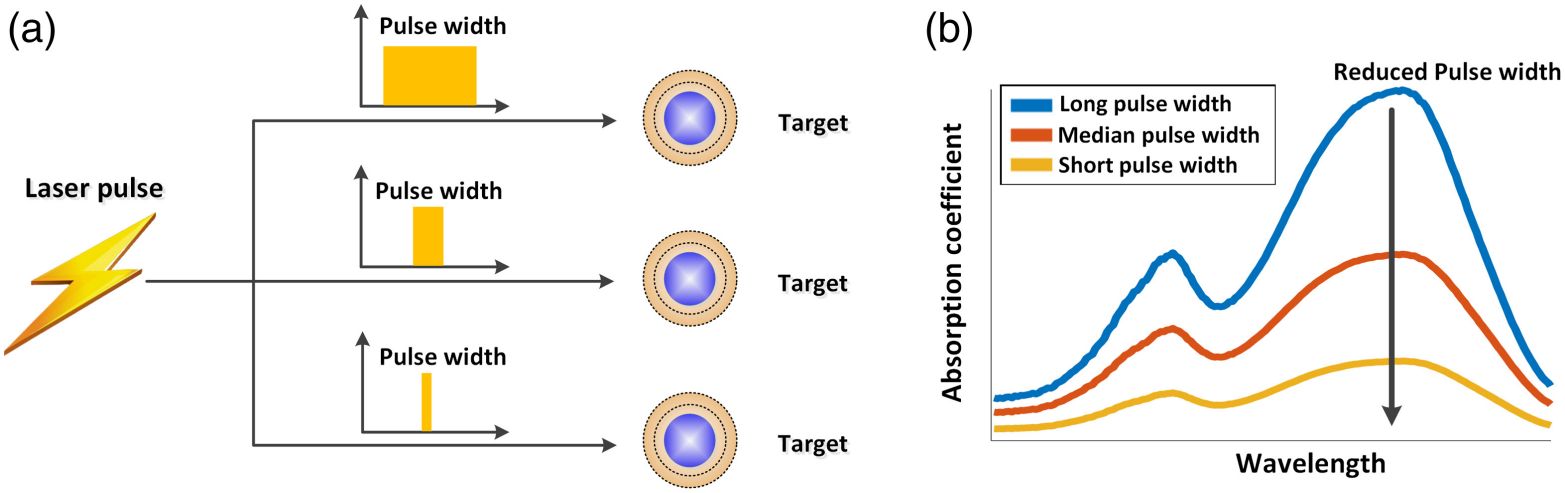
Schematic illustration of the relationship between absorption saturation and pulse duration. (a) A reduction in laser pulse duration leads to (b) a decrease in the absorption coefficient, thereby triggering absorption saturation.

This section explores the relationship between the pulse duration and absorption saturation. Previous studies have demonstrated that the absorption saturation can be elicited by manipulating the laser pulse duration.^2^^,^^24^^,^^95^^–^^98^ Generally, the absorption coefficient $\mu_{a}$ is given by^99^

$$\mu_{a}(I)=\mu_{a0}(\frac{1}{1+I/I_{\mathrm{sat}}}),$$ (6)

where $\mu_{a0}$ denotes the initial values of the absorption coefficient when the laser is not applied. I indicates the laser intensity, and $I_{\mathrm{sat}}$ the saturation intensity of an absorber. $I_{\mathrm{sat}}$ is an inherent property of an absorber, the definition of which can be found in Refs. 2 and 99. According to Eq. (6), with a lower laser intensity ($I\ll I_{\mathrm{sat}}$), $\mu_{a}(I)=\mu_{a0}$ and no saturation occurs. When the intensity approaches the value of $I_{\mathrm{sat}}$, the absorption coefficient $\mu_{a}(I)$ decreases to half of its original value ($\mu_{a0}$). The laser intensity is given as

$$I=F/\tau_{\text{laser}},$$ (7)

where $\tau_{\text{laser}}$ is the laser pulse width and F is the optical fluence. Therefore, the absorption saturation can be triggered using either a short pulse width $\tau_{\text{laser}}$ or a higher optical fluence F. Figure 9 illustrates how the modulation of pulse duration can lead to absorption saturation.

**Fig. 10:**
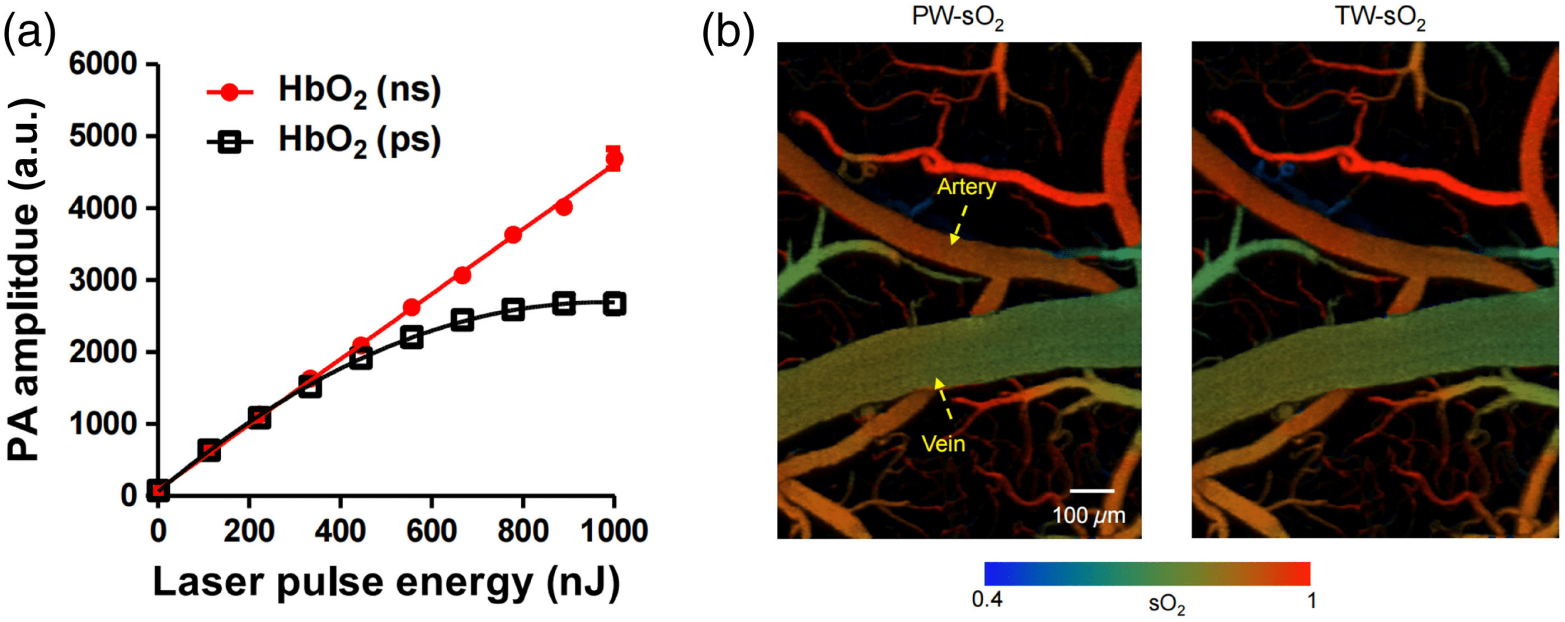
(a) PA signal of oxy-hemoglobin (${\mathrm{HbO}}_{2}$) as a function of the laser pulse energy with a duration of 3 ps and 3 ns. The beam focus in this study remains unchanged; hence, the x-axis essentially indicates the increase in the optical fluence F. The comparison of the two curves indicates that the absorption saturation is more easily induced by picosecond excitation. (b) The comparison of ${\mathrm{sO}}_{2}$ measurements in a major cortical artery–vein pair, using the pulse-width-based method ($\mathrm{PW}\text{-}{\mathrm{sO}}_{2}$) and two-wavelength-based method ($\mathrm{TW}\text{-}{\mathrm{sO}}_{2}$). Figures reproduced with permission from Ref. 38.

By combining Eqs. (2), (6), and (7), we obtain the expression of the PA signal amplitude

$$\mathrm{PA}=k\Gamma \eta_{\mathrm{th}}\mu_{a0}F\frac{1}{1+\frac{F}{\tau_{\text{laser}}I_{\mathrm{sat}}}}=k\Gamma \eta_{\mathrm{th}}\mu_{a0}F\omega ,$$ (8)

where $\omega =\frac{1}{1+\frac{F}{\tau_{\text{laser}}I_{\mathrm{sat}}}}$ is considered to be a nonlinear factor between the PA amplitude and the optical fluence F. When a long pulse duration $\tau_{\text{laser}}$ or a low optical fluence F is utilized, the nonlinear factor ω can be considered negligible; thus, the PA signal demonstrates a linear relationship with the optical fluence F. Conversely, when the pulse width $\tau_{\text{laser}}$ is decreased or a high optical fluence F is employed, the nonlinear factor ω becomes nonnegligible, leading to a nonlinear dependence between the PA signal and the optical fluence F. Figure 10(a) illustrates the dependence of the absorption saturation on the laser pulse duration. By reducing the pulse duration, for example, to picoseconds,^38^ the degree of nonlinearity intensifies, leading to a nonlinear curve between the PA signal and optical fluence, as shown in Fig. 10(a) (black line). The x-axis of Fig. 10(a) essentially indicates the increase in the optical fluence F because the beam focus in Ref. 38 remains unchanged. When the pulse duration is relaxed to nanoseconds, the nonlinear effect becomes negligible, and a linear relationship between the PA signal and optical fluence is maintained [Fig. 10(a), red line]. These results validate the crucial role of the laser pulse duration $\tau_{\text{laser}}$ in determining the occurrence of absorption saturation-based nonlinearity.^2^

The manipulation of the pulse duration to induce absorption saturation offers new opportunities for important utilizations, such as the single-wavelength functional imaging of oxygen saturation (${\mathrm{sO}}_{2}$).^38^^,^^98^ The absorption saturation introduces new dimensions, namely, the pulse duration $\tau_{\text{laser}}$ and the optical fluence F, to absorption spectroscopy, $\mu_{a}=\mu_{a}(\tau_{\text{laser}},F,\lambda )$). This new dependence of $\mu_{a}$ on the pulse duration $\tau_{\text{laser}}$ indicates that the multiple-pulse-duration $\left(\tau_{\text{laser}}\right)$ approach^38^^,^^98^ can be used to produce multiple equations for computing ${\mathrm{sO}}_{2}$, where two unknowns exist, i.e., the concentration of oxy- and deoxy-hemoglobins, i.e., $N_{{\mathrm{HbO}}_{2}}$ and $N_{\mathrm{HbR}}$. The validity of this approach was confirmed by imaging ${\mathrm{sO}}_{2}$ in a major cortical artery–vein pair [Fig. 10(b), left], and the results agreed with the multiwavelength strategy [Fig. 10(b), right]. Compared with the conventional multiple-wavelength approach,^24^^,^^100^^–^^107^ this single-wavelength strategy [Fig. 10(b), left] eliminates the need for wavelength-dependent fluence compensation and enhances the imaging speed by eliminating the necessity for wavelength switching.

### 3.5 Decoupling of Bipolar Signal

**Fig. 11:**
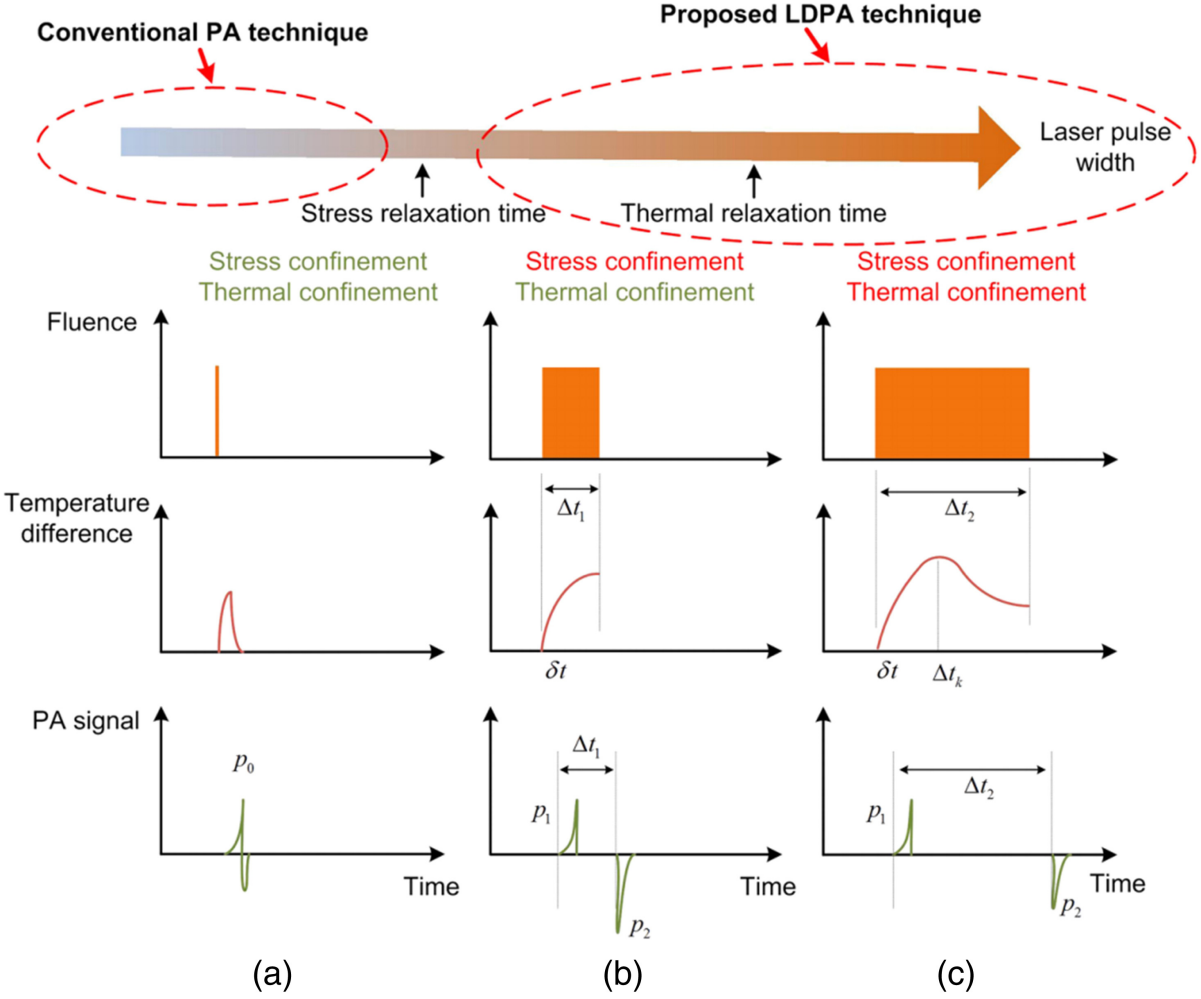
Principle illustration of the temporal decoupling of bipolar PA signals. The first row shows the laser pulse duration, and the second and third rows show the temperature change and PA signal waveform, respectively. The case in which (a) both thermal and stress confinement are satisfied, $\tau_{\text{laser}}<\tau_{s}<\tau_{h}$, (b) only thermal confinement is satisfied $\tau_{s}<\tau_{\text{laser}}<\tau_{\mathrm{th}}$, and (c) both thermal and stress confinements are unsatisfied, $\tau_{s}<\tau_{\mathrm{th}}<\tau_{\text{laser}}$. Figures reproduced with permission from Ref. 39.

**Fig. 12:**
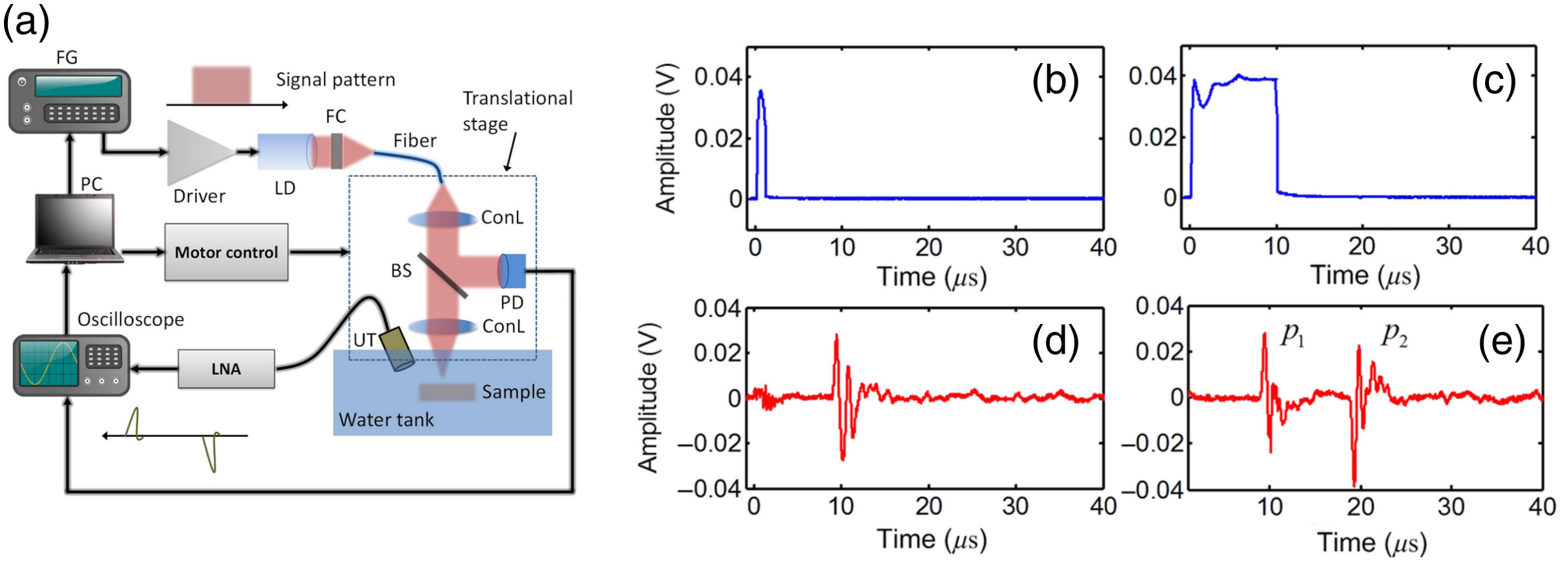
(a) Illustration of the experimental setup. (b), (c) Two laser pulse widths (1 and 10  μs) detected by a photodiode. (d), (e) The measured PA signals showing the temporal separation of a bipolar signal as the pulse width increases to 10  μs. Figures reproduced with permission from Ref. 39.

The manipulation of pulse duration plays a pivotal role in separating the bipolar PA signal.^39^ Generally, a short laser pulse satisfying both stress and thermal confinements typically generates a bipolar PA signal [Fig. 11(a)] due to the thermoelastic expansion and contraction of the absorbers. However, extending the laser pulse width beyond the stress relaxation time allows for the decoupling of bipolar PA signals [Figs. 11(b), and 11(c)]. The temporal separation of the bipolar signal is dependent on both the pulse width and pulse profile. Specifically, the square-shaped laser profile facilitates the separation of bipolar signals due to the sharp rise and fall of temperature induced by this profile,^39^^,^^108^ as shown in Figs. 11(b) and 11(c), first row]. Conversely, when employing a Gaussian-shaped profile, no discernible separation is observed.^108^ In terms of pulse width, increasing the laser pulse width beyond the stress relaxation time gradually separates the single bipolar PA signal into two independent but correlated PA signals. The first pulse, $p_{1}$, represents the expansion-induced positive PA signal, and the second pulse, $p_{2}$, represents the contraction-induced PA signal with a phase-inverted waveform. Generally, $p_{2}$ exhibits a higher amplitude than $p_{1}$ due to greater light energy deposition in the object [Fig. 11(b)]. However, with further increases in the laser pulse width beyond the thermal relaxation time, the signal amplitude of $p_{2}$ begins to decrease [Fig. 11(c)] due to thermal diffusion during prolonged laser illumination. The validity of this signal separation concept was confirmed through experiments involving the tuning of the laser pulse width of a CW laser (Fig. 12), and the outcomes are in line with the theoretical predictions.

This phenomenon helps achieve two nonlinearly correlated PA signals, i.e., the difference between the two signals results in a nonlinear PA signal showing higher order dependence on the optical fluence (F). Based on the nonlinearity PA rules, as outlined in previous studies^2^^,^^29^ that examined how the nonlinear mathematical structure governs its utilization, it can be inferred that this pulse-duration-dependent nonlinearity holds potential for interesting applications such as super resolution achievement^2^ and PA-assisted wavefront shaping.^29^

### 3.6 PT to PA Effect

The modulation of the pulse width in the time domain may alter the conversion process and ratio from the PT to PA effect. Short laser pulses confine energy within the optical zone, which maximizes the PT–photomechanical conversion/interaction.^35^ In such cases, the conversion ratio of the deposited optical energy into acoustic waves through the thermoelastic mechanism is maximized. This indicates that the PA effect plays the dominating role with minimal heat conduction effects in the optical zone of tissue within the pulse duration. However, as the pulse duration increases, heat transfer occurs between the optical absorption zone and its adjacent structures. This is the regime in which the thermal conduction effect starts becoming significant and the PA effect is decreased, owing to the fact that the conversion ratio from the deposited optical energy to the acoustic energy decreases. When the laser pulse duration significantly exceeds the thermal relaxation time, a substantial “heat leakage” occurs between the region of photon deposition and its neighboring structure, leading to temperature increases both within and outside the optical absorption zone on a macroscopic scale. In such instances, the heat conduction effect becomes predominant.

**Fig. 13:**
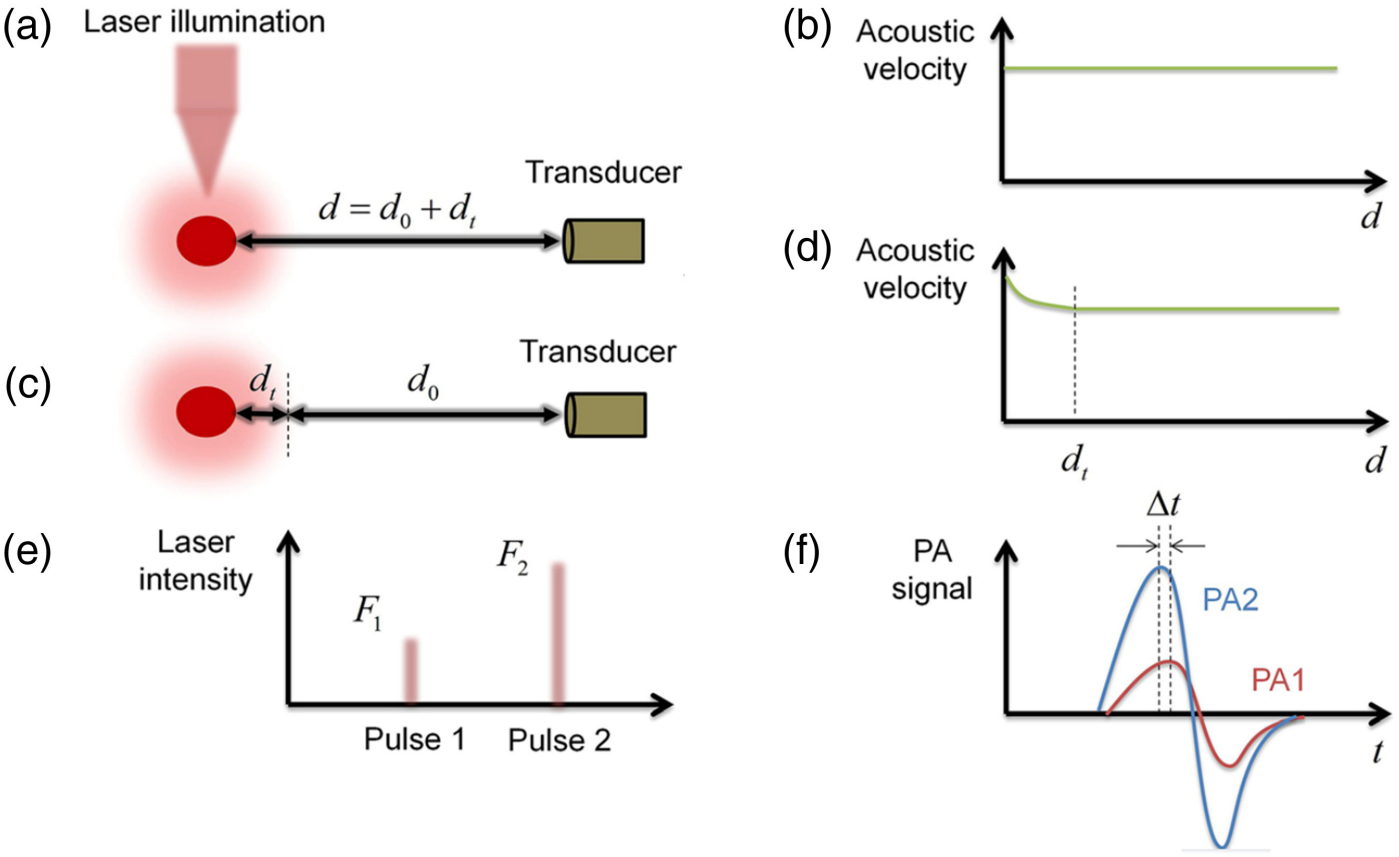
Principle illustration of the phase-domain PA sensing. (a) The first pulse follows the conventional PA propagation because (b) the speed of sound is constant along the PA propagation distance. (c) The propagation of the second pulse is influenced by the first pulse because (d) the speed of sound is influenced by the thermal diffusion region induced by the first laser illumination. (e) The schematic of the two consecutive laser pulses. (f) The phase difference (i.e., time delay) between two consequent laser pulses can be used to represent the amplitude of the first PA signal. Figure reproduced with permission from Ref. 109.

Generally, the thermal diffusion (heat leakage) during laser pulse duration should be avoided to maximize the excitation efficiency of the pressure wave. However, in some cases, the “heat leakage” can be advantageous. For instance, in the technique of phase-domain PA sensing,^109^ the absolute PA signal amplitude is transformed into the signal phase by utilizing the local temperature information of the thermal diffusion region. To be more specific, this study employed two consecutive laser pulses (Fig. 13), and the phase difference (i.e., time delay, Δt) between them can be used to represent the amplitude of the first PA signal. This is because the time delay (Δt), which depends on the sound speed field, is determined by the temperature field induced by the thermal energy absorbed from the first pulse at the time of the second laser excitation. As a result, the signal amplitude is successfully transformed to the signal phase. However, the measurement accuracy of this technique is constrained by the small-scale time delay of the signal reaching UST between the two signals, which is determined by the limited thermal diffusion region (d1), as depicted in Fig. 13(c). Interestingly, the “heat leakage” arising from the violation of thermal confinement may offer potential to address this problem. By tuning the laser pulse to a long duration, the thermal diffusion region (d1) can be extended to a larger zone via the considerable heat conduction between the optical zone and its adjacent structures. This may help guarantee a sufficient thermal diffusion region (d1) and the time delay between two signals, thereby facilitating the measurement accuracy of this technique. In the future, the manipulation of the pulse duration may be explored for the different utilizations/applications of the PA technique in which thermal diffusion is desirable and heat conduction can be utilized effectively.

## 4 Conclusions

In this paper, we discussed and analyzed how the pulse duration alters the mechanisms of optical-acoustic interaction and how it affects the performance of PA imaging. Moreover, we analyzed how the duration of radiation exposure governs the mechanism of PT-PA interaction and triggers physical phenomena, such as absorption saturation and the decoupling of bipolar PA signals. The conclusions are summarized in Table 2 and are detailed below.

**Table 2:** Effect of the light pulse duration on characteristics of PA technique/imaging.

	Short pulse duration	Long pulse duration
Signal amplitude	High SNR with minimal heat conduction and pressure relaxation when both the thermal and stress confinements are met.	Low SNR with nonnegligible heat conduction and pressure relaxation.
Lateral resolution (OR-PAM)	Determined by optical diffraction limit (0.5 λ/NA) when the thermal confinement is met.	Determined by both the optical diffraction limit (0.5 λ/NA) and the laser pulse duration $\tau_{\text{laser}}$, as shown in Fig. 4(a).
Lateral resolution (AR-PAM)	Determined by the center frequency and the numerical aperture of the UST, i.e., $0.71c/f_{c}\mathrm{NA}$. This relationship generally holds true for a short laser pulse (several ns or smaller) because it triggers acoustic waves with a center frequency (hundreds of MHz or above) typically higher than that of UST (generally tens of MHz). Thus, the UST’s center frequency is more restrictive and determines the lateral resolution.	Determined by the center frequency, the numerical aperture of the UST, and the laser pulse duration $\tau_{\text{laser}}$. Note that a long laser pulse (tens of ns or longer) can cause a decreased center frequency compared with that of the UST (generally tens of MHz).
Lateral resolution (PACT)	Determined by the UST’s element aperture size and the bandwidth.^88^ This statement generally holds true for a short laser pulse (several ns or smaller) because it triggers acoustic waves with a bandwidth (hundreds of MHz or above) generally broader than that of UST (generally tens of MHz). Thus, the UST’s bandwidth is more restrictive and determines the axial resolution.	Determined by the UST’s element aperture size and the bandwidth, and the laser pulse duration $\tau_{\text{laser}}$. Note that a long laser pulse (tens of ns or longer) can cause a narrower bandwidth compared with that of the UST (generally tens of MHz).
Axial resolution (OR-PAM, AR-PAM, and PACT)	Determined by the bandwidth of the UST, i.e., 0.88c/Δf. This relationship generally holds true for a short laser pulse (several ns or smaller) because it triggers acoustic waves with the bandwidth (hundreds of MHz or above) generally broader than that of UST (generally tens of MHz). Thus, the UST’s bandwidth is more restrictive and determines the axial resolution.	Determined by both the bandwidth of the UST and the pulse duration $\tau_{\text{laser}}$. Note that a long laser pulse (tens of ns or longer) can cause a narrower bandwidth compared with that of the UST (generally tens of MHz).
Absorption saturation-based nonlinearity	Absorption saturation occurs, and it introduces new dimensions to absorption spectroscopy (i.e., $\mu_{a}=\mu_{a}(\tau_{\text{laser}},F,\lambda )$). This new dependence of $\mu_{a}$ on the optical fluence F and pulse duration $\tau_{\text{laser}}$ yields the nonlinear PA effect.	No absorption saturation occurs. The absorption coefficient remains a monotropic function of the optical wavelength, i.e., $\mu_{a}=\mu_{a}(\lambda )$; thus, a linear PA correlation is maintained according to Eq. (2).
Temporal separation of the bipolar signal	No separation of bipolar signal occurs when the stress confinement is met, i.e., $\tau_{\text{laser}}<\tau_{s}$. The PA bipolar signal caused by the thermoelastic expansion and contraction are tightly coupled.	The separation of the bipolar signal occurs when a square-shaped pulse is employed with a duration exceeding the stress relaxation time, i.e., $\tau_{\text{laser}}>\tau_{s}$. This facilitates the manifestation of a nonlinear PA effect via the signal differential.
PT-PA interaction	Direct coupling of heat energy to vibrational modes,^35^ emphasizing the role of the PA effect when the thermal confinement is met.	Vibrational modes decrease as heat conduction occurs between the optical region and its neighboring structure, highlighting the thermal effect induced by the optical absorption.

(1) *Signal Amplitude*. The decrease in the optical excitation duration helps satisfy both thermal and stress confinements. This guarantees that the optical absorption zone behaves as an adiabatic and isochoric system [Fig. 2(b)], thereby ensuring no energy leakage during laser illumination, either through heat conduction or the work done by stress. Consequently, the conversion of absorbed optical energy into acoustic energy is maximized when it reaches the stress relaxation time, thereby enhancing the SNR of PA imaging.

(2) *Lateral Resolution (OR-PAM)*. The decrease in pulse duration helps confine the energy within the optical zone ($d_{c}$ is shorter than the system $R_{\text{lateral}}$). By contrast, the increase in pulse duration alters the thermal diffusion length ($d_{\mathrm{th}}$) to extend beyond the optical zone. In such cases, neighboring structures outside the illuminated region also produce PA waves due to heat conduction, thereby decreasing the lateral resolution in OR-PAM.

(3) *Lateral Resolution (AR-PAM and PACT)*. The lateral resolution of AR-PAM and PACT is influenced by the center frequency and bandwidth of the detected PA signals. A longer duration for the laser pulse triggers lower frequency content in the acoustic wave, leading to a decreased center frequency and narrower bandwidth compared with that of the UST. This, in turn, decreases the lateral resolution of AR-PAM and PACT.

(4) *Axial Resolution (OR-PAM, AR-PAM, and PACT)*. The axial resolution of PA imaging in various implementations is determined by the bandwidth of the detected PA signals. Increasing the pulse width in the time domain causes a downshift in the acoustic frequency spectrum of the acoustic wave, leading to a narrower bandwidth compared with that of the UST. Consequently, this leads to a reduction in the axial resolution of PA imaging.

(5) *Absorption Saturation*. The manipulation of the laser pulse duration can trigger absorption saturation [Eqs. (6) and (7)], providing a valuable tool for achieving single-wavelength functional imaging of ${\mathrm{sO}}_{2}$. The absorption saturation introduces new dimensions to absorption spectroscopy (i.e., $\mu_{a}=\mu_{a}(\tau_{\text{laser}},F,\lambda )$). By utilizing multiple pulse durations $\tau_{\text{laser}}$, it is possible to derive multiple equations for computing ${\mathrm{sO}}_{2}$, thereby obviating the need for multiple wavelengths and addressing the challenges associated with wavelength switching and wavelength-dependent fluence compensation in deep tissue imaging.

(6) *Decoupling of Bipolar Signal*. The pulse duration plays a pivotal role in decoupling the bipolar PA signal resulting from the thermoelastic expansion and contraction in the time domain [Fig. 11(b), third row]. By extending the width of the laser pulse to exceed the stress relaxation time with a square-shaped pulse,^39^ the two correlated PA signals can be effectively separated. This phenomenon enables the manifestation of a nonlinear PA effect through the signal differential. This pulse-width-dependent nonlinear effect provides opportunities for interesting applications such as super resolution and PA-assisted wavefront shaping.

(7) *PT-PA Interaction*. Manipulating the pulse duration offers precise control over the PT-PA interaction, allowing for the manipulation of the balance between the vibrational mode (PA effect) and thermal conduction in biomedical imaging. Shortening the pulse duration enhances the direct coupling of heat energy to vibrational modes, emphasizing the role of the PA effect. Conversely, lengthening the pulse duration facilitates heat conduction between the optical region and its neighboring structure, highlighting the thermal effect induced by the optical absorption. This manipulation of the pulse duration offers a means to strengthen the PA effect in conventional imaging applications or highlight heat conduction, as is the case in phase-domain PA sensing,^109^ in which thermal effects can be effectively utilized.

In conclusion, this study contributes to the understanding of the physical mechanisms governing pulse-width-dependent PA techniques and offers insights into the nonlinear behavior of the PA signal. By gaining insight into the mechanism behind the influence of the laser pulse, we can trigger the pulse-with-dependent physical phenomena for specific applications, enhance PA imaging performance in biomedical imaging scenarios, or modulate PT-PA conversion by precisely tuning the pulse duration. Short pulse durations are generally advantageous in conventional PA imaging as they can boost the SNR and spatial resolution. However, longer pulse durations also offer distinct advantages. For example, they facilitate the separation of bipolar PA signals, enabling the implementation of nonlinear PA techniques through signal differentiation. In addition, longer pulses can amplify heat conduction effects, leveraging thermal phenomena for specific applications.

## Data Availability

Data underlying the results may be obtained from the authors upon reasonable request.
